# Sinomenine hydrochloride inhibits breast cancer metastasis by attenuating inflammation-related epithelial-mesenchymal transition and cancer stemness

**DOI:** 10.18632/oncotarget.14593

**Published:** 2017-01-10

**Authors:** Xiao Li, Pingping Li, Chao Liu, Yu Ren, Xiaojiang Tang, Ke Wang, Jianjun He

**Affiliations:** ^1^ Department of Breast Surgery, First Affiliated Hospital of Xi'an Jiaotong University, Xi'an 710061, P.R. China; ^2^ Translational Medical Center, First Affiliated Hospital of Xi'an Jiaotong University, Xi'an 710061, P.R. China; ^3^ Department of Vascular Surgery, First Affiliated Hospital of Xi'an Jiaotong University, Xi'an 710061, P.R. China

**Keywords:** sinomenine hydrochloride, breast cancer, metastasis, EMT, CSC

## Abstract

Sinomenine hydrochloride (SH) has been investigated for its anti-tumor growth effect. We have previously reported that SH inhibited breast cancer cell proliferation via MAPKs signaling. However, whether SH could inhibit tumor metastasis has not been fully explored. In this study, we found that SH suppressed the metastasis potential of breast cancer cells. The wound healing and transwell assays showed that SH inhibited the migration and invasion ability of both 4T1 and MDA-MB-231 breast cancer cells. The orthotopic mouse model of 4T1 and the experimental mouse model of MDA-MB-231-luc (MDA-MB-231 cell line expressing firefly luciferase) demonstrated that SH treatment inhibited breast cancer metastasis by inhibiting epithelial–mesenchymal transition (EMT) and cancer stem cell (CSC) properties without obvious hepatotoxicity and renal toxicity. We also found that SH decreased spleen volume and weight in both mouse models, especially in the 4T1 mouse model. IL-6, a strong inflammatory factor causing EMT, was remarkably reduced. Overall, this anti-metastasis effect of SH could be possibly caused by attenuating inflammatory reaction, which led to inhibition of EMT and CSC characteristics of breast cancer cells. This study, together with our previous one, provides more evidence of SH as a potential drug for breast cancer therapy.

## INTRODUCTION

Sinomenine (Supplementary Figure 1A) is an abstract isolated from the traditional Chinese herb *Sinomenium acutum* Rehd. *et* Wils. (Fam. Menispermaceae). Previous studies have reported the pharmacological effects of sinomenine, including anti-arthritis effect [1], anti-inflammatory [2], anti-cancer effect [3] and so on. Recently, the anti-proliferation and anti-cancer effects of sinomenine have drawn considerable attention. Sinomenine inhibited proliferation and induces apoptosis of NCI-H460 cells in a dose-dependent manner through the mitochondrial pathway [4]. Lu et al. found that sinomenine increased p21, decreased the Bcl-2/Bax ratio, promoted the release of Cytochrome c and Omi/HtrA2 from the mitochondria into the cytoplasm and induced the cleavage of caspase-3 and -9 [3]. Our previous study also showed that sinomenine hydrochloride (SH) (Supplementary Figure 1B), a hydrochloride chemical form of sinomenine which is water soluble, arrested cell population at G1 phase, caused cell apoptosis and induced ATM/ATR-Chk1/Chk2-mediated DNA damage in breast cancer cells through regulation of MAPKs pathways [5]. During our study of the anti-proliferation effect of SH [5], we also found that SH could inhibit the invasion and metastasis ability of breast cancer cells. However, there is no study fully investigated the anti-invasion and anti-metastasis effects of SH on breast cancer cells and explored the potential mechanisms. So we carried out experiments to explain the anti-invasion and anti-metastasis effect of SH on breast cancer cells.

Breast cancer is one of the most common malignant diseases in western women with an estimated rate of 29% of all the new cancers in women in 2016 [6]. According to the American Cancer Society, one in eight women in the United States will develop breast cancer in her lifetime [7]. Although more and more effective strategies have improved patients’ survival, breast cancer metastasis is still the major reason for morbidity and mortality in breast cancer patients [8–10]. Therefore, it is necessary to search for novel effective adjuvant agents to treat metastatic breast cancer.

Epithelial to mesenchymal transition (EMT) is an essential process in many human activities such as embryonic development, tissue remodeling and wound healing [11, 12]. Studies have shown that EMT, accompanied by loss of polarity and gain of motility, plays an important role in the process of metastasis [13, 14]. EMT is also involved in the generation of cancer stem cells (CSCs) [13, 15, 16]. CSC is a small subpopulation of cancer cells with the ability of self-renewal, contributing to cancer metastasis and chemoresistance [17, 18]. Studies show that EMT induction and post-EMT maintenance rely on microenvironment and cytokines [19, 20]. Thus, inhibiting the inflammatory cytokine production would suppress both the EMT process and the generation of CSCs, leading to more effective therapy.

In this study, we used mouse breast cancer cell line 4T1 and human breast cancer cell line MDA-MB-231 to investigate the anti-metastasis effect of SH and the potential mechanisms. The *in vitro* experiments showed that SH could inhibit the migration and invasion ability of breast cancer cells. The *in vivo* mouse models also demonstrated that SH inhibited distant metastasis of breast cancer cells by reversing the EMT transition and suppressing the CSC phenotypes. SH also inhibited spleen weight and volume, as well as inflammatory factor IL-6, a very important inducing and maintenance factor in EMT and CSC, in both animal models. The inhibition effect of SH on EMT and CSC generation on breast cancer cells could be caused by its anti-inflammatory effect.

## RESULTS

### SH blocked breast cancer metastasis in 4T1 orthotopic metastatic mouse model

**Figure 1:**
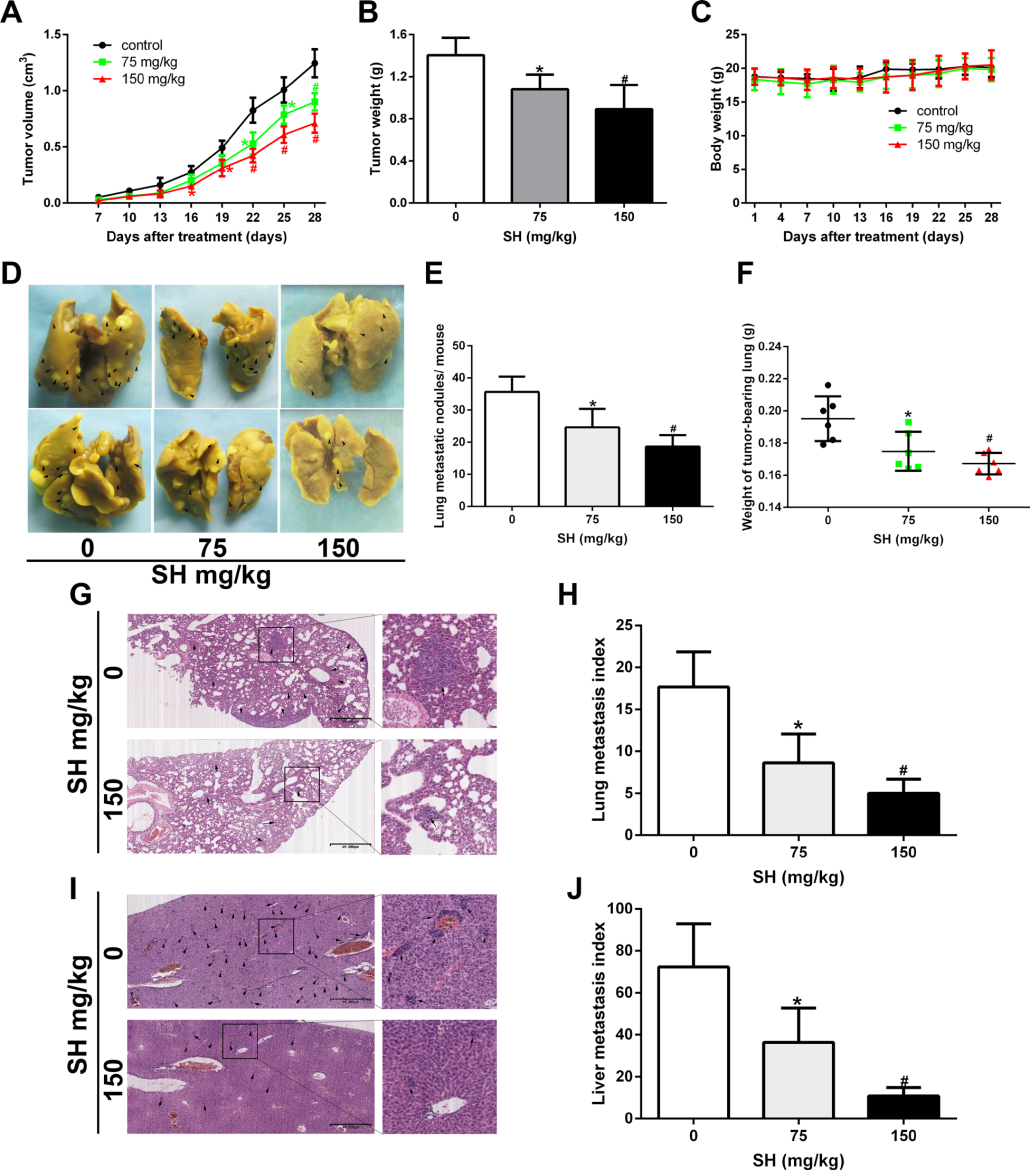
SH inhibited breast cancer metastasis in a 4T1 metastatic mouse model BALB/c mice bearing 4T1 mouse breast homograft were treated with physiologic saline or different doses of SH. (**A**) Time course of primary tumor volume. (**B**) Primary tumor weight. (**C**) Body weight. (**D**) Representative lung pictures fixed by Bouin's solution. Metastatic foci on the gross tissue were marked with black arrows. (**E**) Lung metastatic nodules per mouse. (**F**) Lung weight. (**G**) HE staining of lung specimen and (**H**) lung metastasis index. (**I**) HE staining of liver specimen and (**J**) liver metastasis index. Data are represented as mean ± S.D. of three independent experiments. *P < 0.05, #*P* < 0.01, SH treated group compared with the untreated control group.

4T1 cells are very aggressive murine breast cancer cells which are usually used to make models of late stage breast tumor to investigate the metastatic behavior [21]. 1 × 10^5^ 4T1 cells were injected into the left second mammary fat pad of the mice to establish an orthotopic metastatic mouse model, as the 4T1 cells were obtained from BALB/c mice, they could spontaneously metastasize to secondary foci (lymph node, lung, and liver) from the primary sites. After 4 weeks, SH treatment reduced tumor volume (Figure 1A) and tumor weight (Figure 1B). After organs were removed from the mouse under anesthesia, lung weight was measured. The results showed that the lung weight was 0.1952 g, 0.1748 g and 0.1672 g in the control, 75 mg/kg SH and 150 mg/kg SH groups, respectively (Figure 1F). Then the lungs were fixed and stained in Bouin's solution (Figure 1D) and lung metastatic nodules were counted (Figure 1E). The results of histological examinations of metastasis of the lungs and livers were shown in Figure 1G–1J. Compared with the control group, 150 mg/kg SH treatment reduced lung index to 28.14% and liver index to 14.86%. We then examined the liver function and renal function of the mice to test whether SH treatment would cause obvious toxicity on mice. The results showed that there was not statistical significance between the control group and the SH-treated groups, indicating that SH treatment did not exert obvious toxicity on mice (Supplementary Table 1).

### SH prevented breast cancer metastasis in MDA-MB-231-luc metastatic mouse model

**Figure 2:**
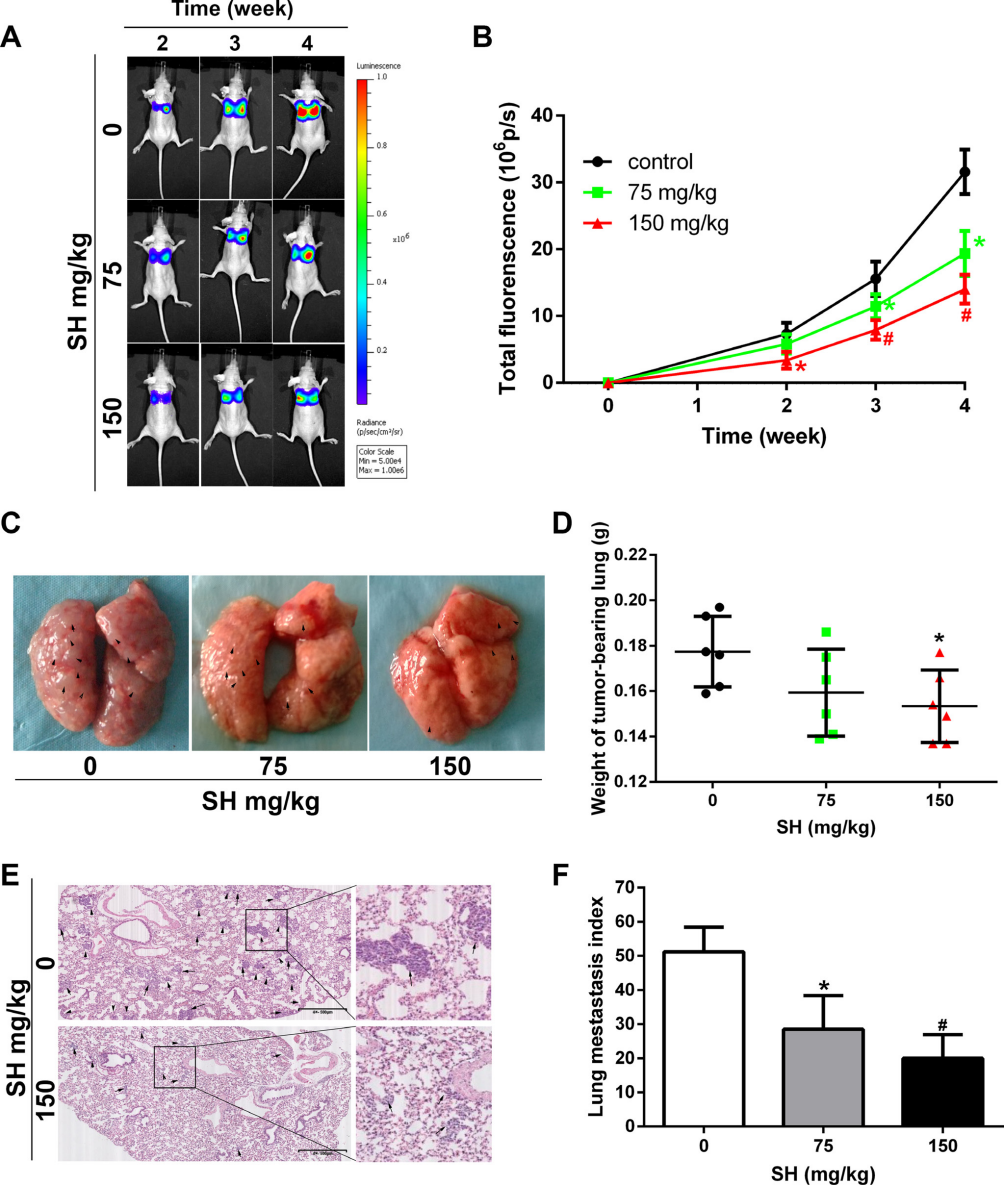
SH reduced pulmonary metastasis in an MDA-MB-231-luc experimental metastatic mouse model BALB/c mice were injected with MDA-MB-231-luc via tail vein. (**A**) Representative bioluminescence images of lung metastasis. Luciferin was injected i.p. and lung metastasis was monitored by a Xenogen IVIS 2000 Imager at the indicated time points. The color scale to the right of the image showed the intensity range of photon flux per second. (**B**) The quantification of total lung fluorescence. (**C**) Representative pictures of gross lung. Black arrows indicated metastatic nodules. (**D**) Lung weight. (**E**) HE staining of lung specimen and (**F**) lung metastasis index. Data are represented as mean ± S.D. of three independent experiments. *P < 0.05, #*P* < 0.01, SH treated group compared with the untreated control group.

To further investigate the effect of SH on breast cancer metastasis, MDA-MB-231-luc cells were injected into the tail vein of nude mice to establish experimental metastasis model. Mouse were injected with physiologic saline, 75 mg/kg SH or 150 mg/kg SH. Lung metastasis was monitored by a Xenogen IVIS 2000 Imager consecutively until four weeks of the treatment (Figure 2A). SH treatment decreased the total lung fluorescence (Figure 2B). As shown in Figure 2C, more nodules could be seen on gross lung picture in the control group. From Figure 2D, we could see that 75 mg/kg and 150 mg/kg SH decreased average lung weight to 0.1593 g and 0.1533 g, respectively, compared with the control group of 0.1774 g (*p* = 0.147 and *p* = 0.048, respectively). Although there was a decrease trend of lung weight in low dose SH treatment, there was no statistical significance. It could be due to the relative small number of the samples. The microscopic lung metastasis was evaluated using the same method as in the 4T1 orthotopic metastasis model. After treatment with physiologic saline, 75 mg/kg and 150 mg/kg SH, the lung index was 51.17 ± 7.25, 28.50 ± 9.88 and 20.00 ± 6.95, respectively. Compared with the control group, 75 mg/kg SH treatment reduced lung index to 55.70% and 150 mg/kg SH reduced lung index to 39.09%. Supplementary Figure 2 showed a very small increase in weight in all groups without significant difference between the control group and the treated groups. Supplementary Table 2 demonstrated no significant liver and renal function changes of SH in comparison with physiologic saline. Supplementary Figure 2, together with Supplementary Table 2 suggested the relative safety of SH at the chosen doses.

### SH decreased MMP-9 and increased TIMP-1 and TIMP-2 expression

**Figure 3:**
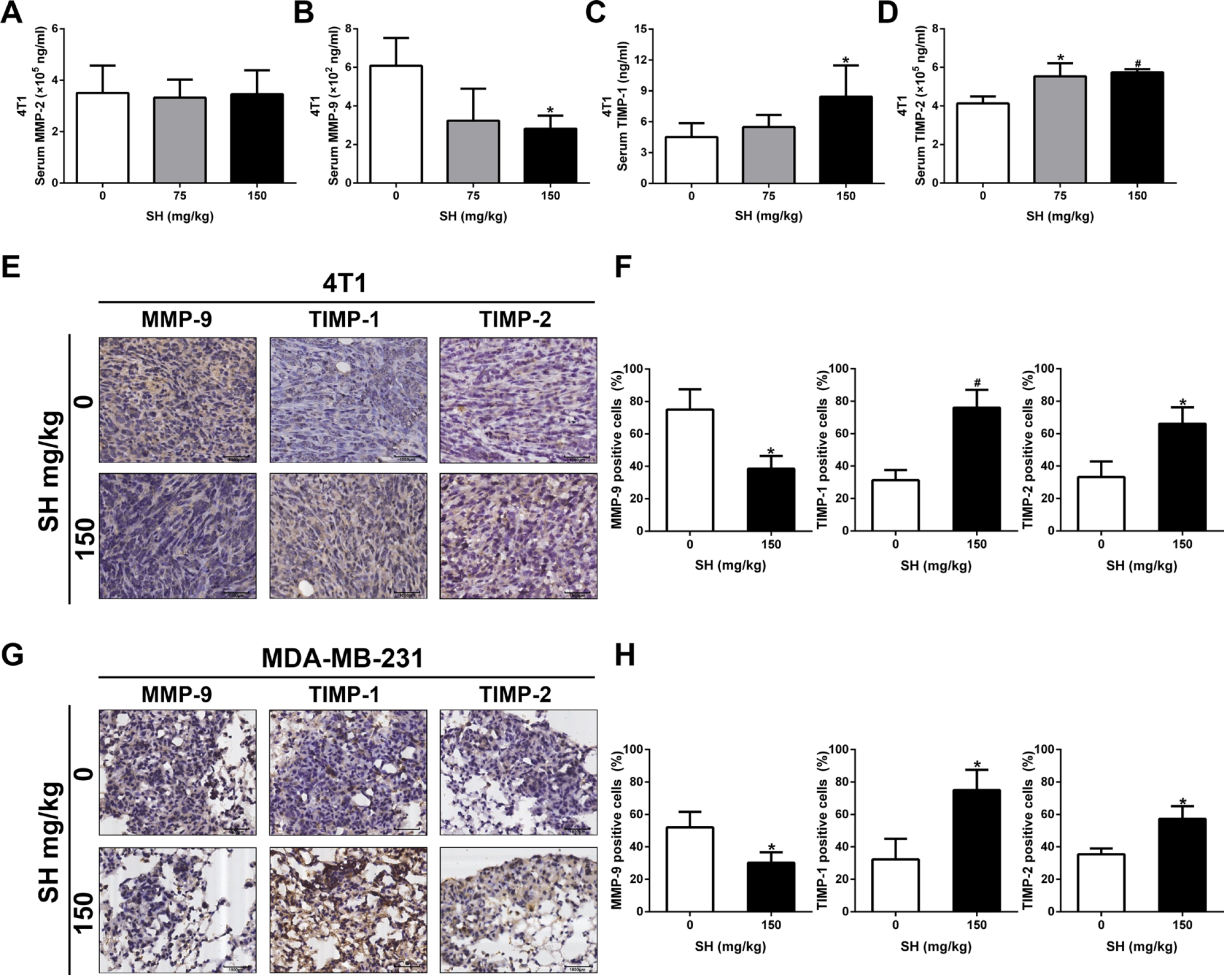
SH decreased MMP-9 and increased TIMP-1, TIMP-2 levels in both mouse model Serum samples and section specimen were used to detect these markers. In the 4T1 mouse model, (**A**) MMP-2, (**B**) MMP-9, (**C**) TIMP-1 and (**D**) TIMP-2 serum levels were tested using ELISA. (**E**) IHC staining and (**F**) quantification of MMP-9, TIMP-1 and TIMP-2 expression in 4T1 tumor specimens. (**G**) IHC staining and (**H**) quantification of MMP-9, TIMP-1 and TIMP-2 expression in MDA-MB-231 lung specimens. Data are represented as mean ± S.D. of three independent experiments. *P < 0.05, #*P* < 0.01, SH treated group compared with the untreated control group.

Cancer metastasis is a complex process including tumor cell adhesion to extracellular matrix (ECM), migration, invasion and other intravascular and extravascular activates [22]. During the invasion and metastasis process, cancer cells need to increase matrix metalloproteinase (MMP) expression and decrease tissue inhibitor of metalloproteases (TIMP) expression to degrade ECM [23, 24]. We first detected the serum levels of MMP-2, MMP-9, TIMP-1 and TIMP-2 in both mouse models by enzyme-linked immunosorbent assay (ELISA). For the 4T1 mouse model, the results showed that SH treatment decreased MMP-9 serum level, while increased TIMP-1 and TIMP-2 serum levels (Figure 3B–3D). However, no significant change of MMP-2 was detected (Figure 3A). For the MDA-MB-231 mouse model, as we detected human breast cancer cell excretion in mouse serum, the level of these markers were very low. Therefore, it required large quantity of serum far beyond the serum volume of the samples, and due to the very low concentrations of these tested markers, the established ELISA standard curve in our preliminary experiments showed that it was not accurate enough to predict the samples at such low concentrations.

We further used IHC to reaffirm the results of MMP-9, TIMP-1 and TIMP-2. In 4T1 tumor section specimens, we found that 150 mg/kg decreased MMP-9 to 38.54% compared with 75% in the control group, increased TIMP-1 to 76.04% compared with 31.25% in the control group, and increased TIMP-2 to 66.15% compared with 33.33% in the control group, respectively. We also could detect changes in MMP-9, TIMP-1 and TIMP-2 in the lung section specimens of MDA-MB-231 experimental mouse model. SH treatment decreased MMP-9 to 30.21% compared with 52.08% in the control group, increased TIMP-1 to 75% compared with 32.29% in the control group, and increased TIMP-2 to 57.29% compared with 35.42% in the control group, respectively. The change of TIMP/MMP meant that SH could inhibit breast cancer cells from degrading ECM, thus preventing metastasis.

### SH impaired migration and invasion ability and inhibited EMT process in breast cancer cells

**Figure 4:**
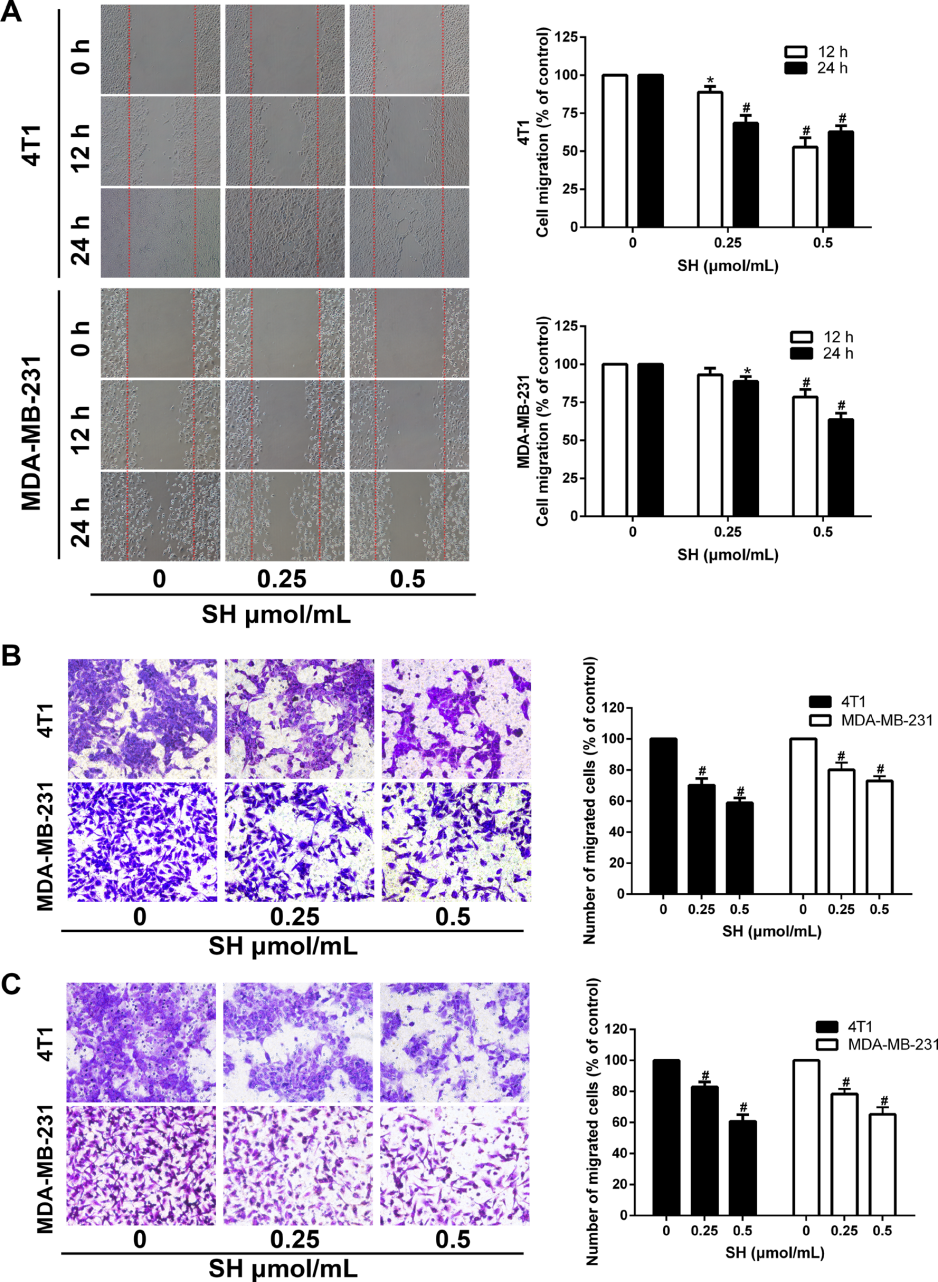
SH impaired the migration and invasion ability of 4T1 and MDA-MB-231 cells (**A**) Confluent cells were scratched and treated with SH for 12 and 24 h. Cells migrating into the scratched area were photographed (×40). Cell migration ability was assessed by migration area. (**B**) 4T1 and MDA-MB-231 cells were cultured with different doses of SH in the upper transwell chambers. After migrating for 24 h, cells on the bottom of the filter were fixed, stained and photographed (×200). The migrated cells were dissolved in 30% acetic acid and measured by a microplate reader at 570 nm. (**C**) SH inhibited the invasion ability of 4T1 and MDA-MB-231 cells. Matrigel was coated on the transwell chambers. After 24 h, the invaded cells were photographed (×200) and quantified by a microplate reader at 570 nm. All data are represented as mean ± S.D. of three independent experiments. *P < 0.05, #*P* < 0.01, SH treated group compared with the untreated control group.

Since EMT is recognized for its role in tumor metastasis [11, 15], we then examined whether SH could reverse the EMT process in breast cancer cells. As EMT process often facilitates cancer cell motility and invasion, we examined the effect of SH on cancer cell migration *in vitro*. Wound healing assay was first used to measure the effect of SH on cancer cell migration. As shown in Figure 4A, after treatment with SH, a clear time- and dose-dependent inhibition was observed. After treatment with 0.25 and 0.5 μmol/mL SH for 24 h, the migration areas were about 68.52% and 62.88% of that of the control group for 4T1, respectively, and 88.79% and 63.68% of that of the control group for MDA-MB-231, respectively.

Transwell migration method was used to confirm the impairment of SH on the migration ability of 4T1 and MDA-MB-231. Treatment with SH at 0.25 and 0.5 μmol/mL significantly impaired the migration ability of 4T1 and MDA-MB-231. The inhibition rates of 0.5 μmol/mL SH for 4T1 and MDA-MB-231 were 59.94% and 72.9%, respectively (Figure 4B) Similar results were found in the matrigel-coated transwell invasion assay. The results showed that 0.5 μmol/mL SH inhibited the invasion ability of 4T1 and MDA-MB-231 to 60.77% and 65.28% of that of the control group, respectively (Figure 4C).

**Figure 5:**
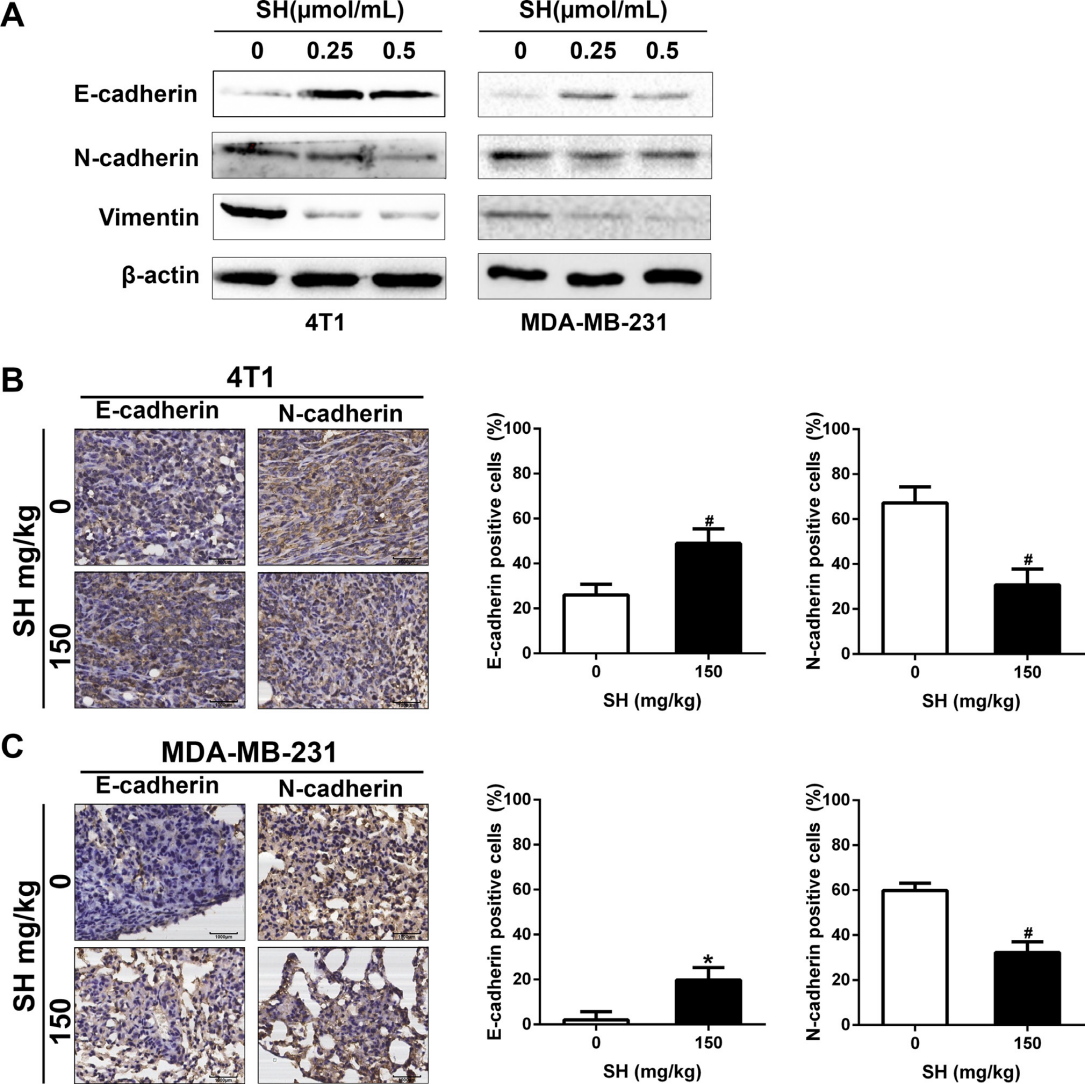
SH reversed EMT process in breast cancer cells (**A**) Proteins involved in EMT process were analyzed by western blot. (**B** and **C**) IHC analysis of EMT markers E-cadherin and N-cadherin in tumor specimens of 4T1 and lung specimens of MDA-MB-231.

We then detected some EMT markers in 4T1 and MDA-MB-231 cell lines *in vitro*. After treatment of SH for 24 h, the western blot results (Figure 5A) showed that SH treatment increased the epithelial marker E-cadherin, and decreased the mesenchymal marker N-cadherin and vimentin in 4T1 cell line. However, the downregulation of N-cadherin was not obvious in MDA-MB-231 cell line which could be due to different response of different cell lines at the same time.

We also detected some markers using samples from mouse models. Figure 5B showed that SH treatment increased E-cadherin from 26.04% in the control group to 48.96% in the 150 mg/kg group of the tumor specimens and from 2.08% to 19.79% of the lung samples. In contrast, SH treatment decreased N-cadherin from 67.13% in the control group to 30.73% in the 150 mg/kg group of the tumor specimens and from 59.90% to 32.29% of the lung samples (Figure 5C). These results suggested the reverse of EMT by SH treatment.

### SH suppressed CSC characteristics in breast cancer cells

**Figure 6:**
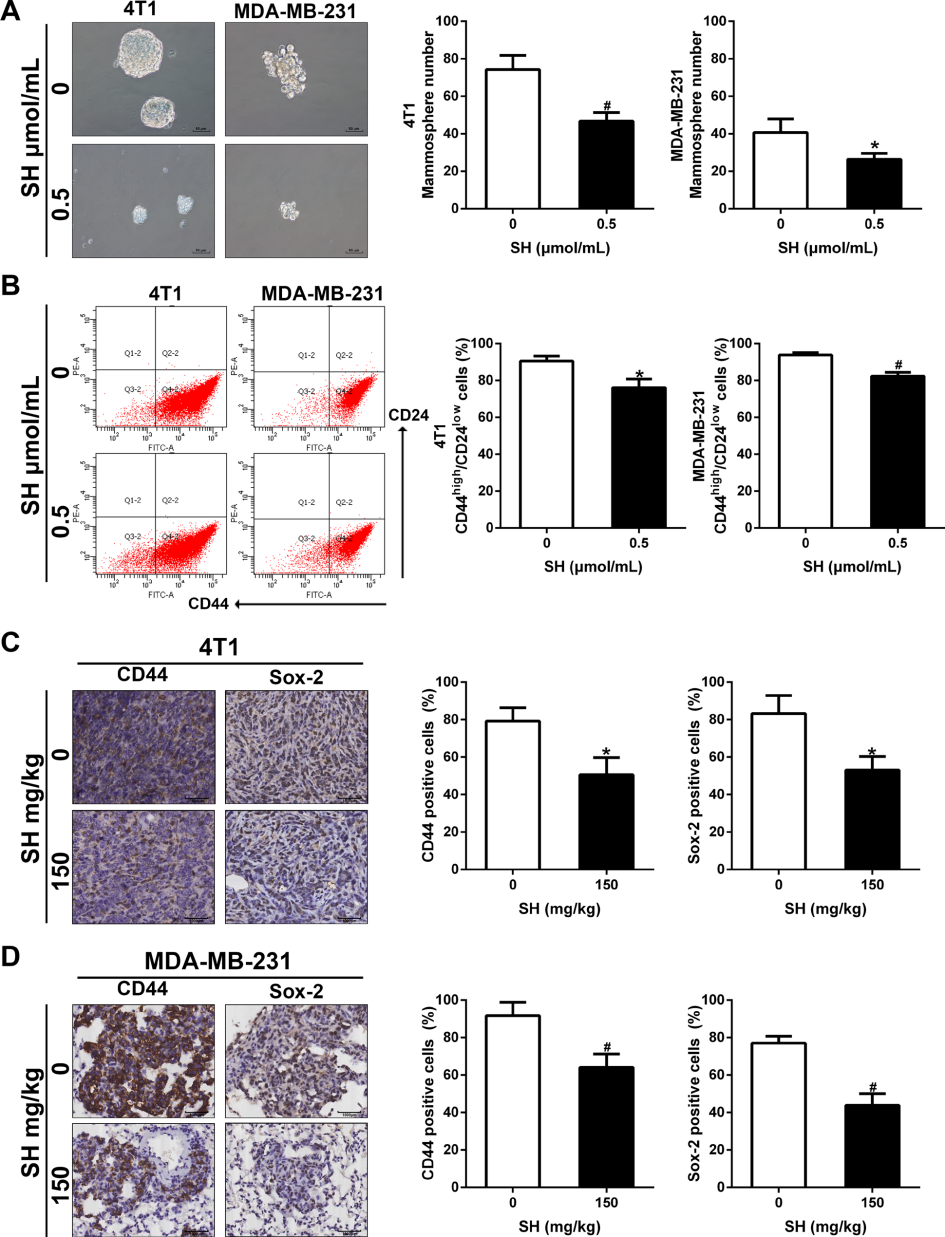
SH inhibited CSC characteristics in breast cancer cells (**A**) Mammosphere formation and its quantification. 4T1 and MDA-MB-231 were treated with SH for 48 h, harvested and seeded on ultra-low attachment culture plates. Mammospheres with diameter > 50 μm were counted on day 10. (**B**) CSC markers CD44 and Sox-2 were analyzed by western blot. (**C** and **D**) IHC analysis of CD44 and Sox-2 in tumor specimens of 4T1 and lung specimens of MDA-MB-231. Data are represented as mean ± S.D. of three independent experiments. *P < 0.05, #*P* < 0.01, SH treated group compared with the untreated control group.

EMT and CSC are interrelated with each other. Evidence have shown that EMT could induce CSC characteristics [16]. We wondered whether inhibition of EMT by SH could contribute to some changes in the CSC characteristics. Mammosphere formation is good a way to assess self-renewal ability of CSCs, involving *in vitro* spheroids formation in the non-adherent culture conditions. The results showed that SH decreased the number of sphenoid colonies of both 4T1 and MDA-MB-231 (Figure 6A). The number of sphenoid colonies of 0 and 0.5 μmol/mL SH treatment was 74.33 and 46.67 for 4T1, respectively (*p* < 0.01). The number of sphenoid colonies of 0 and 0.5 μmol/mL SH treatment was 40.67 and 26.33 for MDA-MB-231, respectively (*p* < 0.05).

CD44 and Sox-2 are both markers of stem cancer cell. We used flow cytometer to assess CD44 changes in MDA-MB-231 and 4T1 cell lines first. Figure 6B showed 0.5 μmol/mL SH decreased expression of CD44 on cell membrane in both cell lines. We then evaluated their expression in tumor samples and lung samples. Figure 6C–6D demonstrated that SH decreased CD44 and Sox-2 in both mouse models, suggesting the inhibition of CSC by SH treatment.

### SH inhibited inflammation response elicited by breast cancer cells

**Figure 7:**
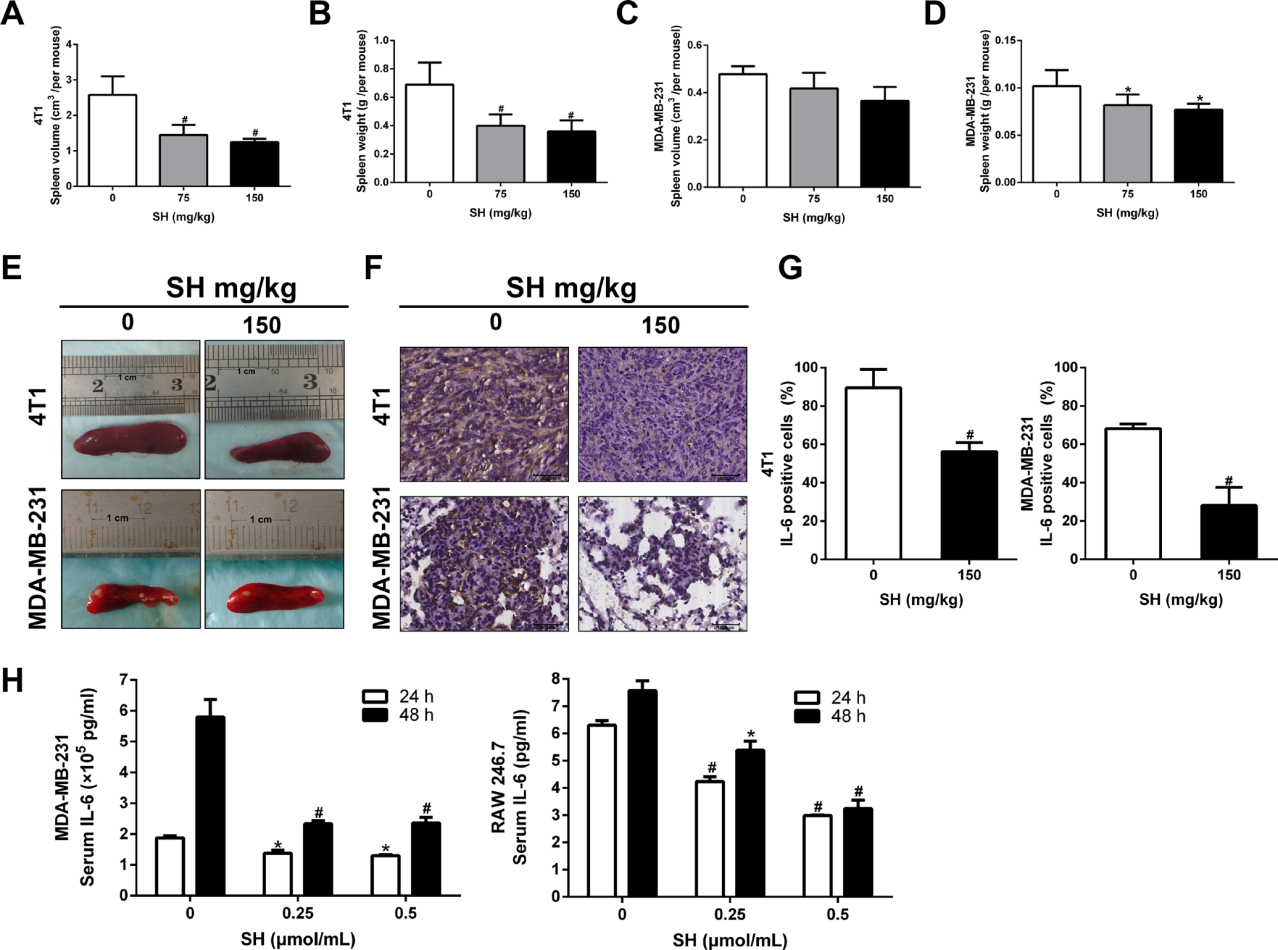
SH attenuated inflammation response elicited by breast cancer cells (**A**) Spleen volumes and (**B**) spleen weight of the control group, 75 mg/kg and 150 mg/kg SH groups in the 4T1 mouse model. (**C**) Spleen volumes and (**D**) spleen weight of the control group, 75 mg/kg and 150 mg/kg SH groups in the MDA-MB-231-luc mouse model. (**E**) Representative spleen pictures. (**F** and **G**) IL-6 expression analysis by IHC. (**H**) IL-6 secretion of cells was tested using ELISA. MDA-MB-231 cancer cells and RAW 246.7 murine macrophages were treated with SH and the supernatant was used to test IL-6 secretion. Data are represented as mean ± S.D. of three independent experiments. *P < 0.05, #*P* < 0.01, SH treated group compared with the untreated control group.

SH is famous for its remarkable anti-inflammation effect in rheumatic arthritis. Most of the previous studies also focused more on its anti-inflammatory effect. EMT is also known for its induction and maintenance by cytokines and inflammatory factors [19, 20]. We wondered whether the inhibition of EMT and CSC was due to the anti-inflammation of SH. Obvious decreases of spleen volume and weight were found in the 4T1 orthotopic mouse model (Figure 7A and 7B). The results showed that the volume of the control group was 2.58 cm^3^, the 75 mg/kg group was 1.45 cm^3^, and the 150 mg/kg group was 1.24 cm^3^. The spleen weight of the control group, the 75 mg/kg and the 150 mg/kg group were 0.6885 g, 0.3993 g and 0.3593 g, respectively. There is significant difference of between the control group and the treatment groups. For the MDA-MB-231-luc mouse model, SH treatment also decreased both the spleen volume and spleen weight (Figure 7C and 7D). However, despite of a decrease trend in spleen volume, there was no significance between the control group and the treatment groups (*p* = 0.278 for control vs 75 mg/kg group; *p* = 0.052 for control vs 150 mg/kg group).

IL-6 is one of the famous factors inducing EMT and also has been shown to be reduced by SH. We then detected the expression of IL-6 inflammatory factors in tumor specimens of 4T1 and lung specimens of MDA-MB-231. Figure 7F and 7G showed that 150 mg/kg SH decreased IL-6 to 89.58% compared 56.25% with the control group in 4T1 tumor specimens. Similar results were found in the MDA-MB-231 lung specimens. IL-6 could be secreted by many inflammatory cells as well as tumor cells. We wondered whether the reduction of IL-6 in mouse samples was a direct effect on tumor cells or was an indirect effect on inflammatory cells. We then detected the effect of SH on IL-6 expression *in vitro* to help us answer this question. IL-6 is mainly secreted by monocytes, macrophages, neutrophils and some types of T cells in body [25]. RAW 246.7 is a murine macrophage cell line that could be cultured *in vitro*. We selected RAW 246.7 as the representative inflammatory cell line as macrophage is one of the main production of IL-6 in human. We found that SH decreased IL-6 secretion in both MDA-MB-231 and RAW 246.7 cells. For 4T1 cell line, however, the concentration of IL-6 was too low to fall into the proper range of the ELISA kit.

## DISCUSSION

Although many preventive methods and diagnostic methods have been made to try to detect breast cancer in early stages, there are still a lot of people diagnosed at late stages or with metastasis. Besides, some patients are found initially sensitive to chemotherapy, but later develop chemoresistance and metastasis. As we know most breast cancer mortality results from cancer relapse with invasion and metastasis [6, 26, 27], therefore, effective decrease or treatment of metastasis is important to improve life quality and increase life survival for breast cancer patients.

Cancer metastasis is a complex biological event including two main parts, namely the intravasation process, in which cancer cells of local tumors invade the basement membrane and circulate through the blood, and the extravasation process, in which the cancer cells escape from the capillary wall and survive at distant organ sites [28]. Our previous studies found that SH could effectively inhibited breast cancer cell proliferation [5]. In this study, we investigated the anti-metastasis effect of SH on breast cancer cells in both mouse models of breast cancer.

Choosing suitable models which could mimic the real metastatic process is the first step to study breast cancer metastasis. In the orthotopic metastatic mouse model, cancer cells need to go through both the intravasation and extravasation processes to initiate tumor formation on a new site. 4T1 murine mammary carcinoma cells were always used for its highly metastatic potential in immunocompetent mice. 4T1 mouse model, a model of late-stage breast cancer [29, 30], was chosen to assess the effect of SH on the whole metastatic process from the primary tumor location to the new site via the hematogenous pathway. We found significant differences between the control group and the SH-treated groups. 75 mg/kg and 150 mg/kg SH both decreased the tumor volume and tumor weight in comparison with the control group. More important, SH treatment inhibited lung weight, indicating the anti-metastatic ability of SH. From a microscopic view, SH reduced both lung and liver metastatic index, demonstrating that SH could inhibit new metastatic foci of breast cancer tumors.

Although 4T1 orthotopic mouse model represents the natural metastatic event more authentically, it has a disadvantage, namely 4T1 is a murine cell line. Thus we still wanted to examine the anti-metastatic effect of SH on human breast cancers. Meanwhile, SH treatment inhibited primary tumor growth which could influence tumor cell metastasis [31]. In the experimental metastatic mouse model, MDA-MB-231-luc human breast cancer cells were introduced directly into the tail vein and passed through the lung microvasculature [22]. This model mainly mimics the extravasation process of metastasis. The cell line was modified with firefly luciferase, allowing us to monitor the lung metastasis non-invasively and dynamically [30]. We found that SH treatment decreased the lung metastasis during the consecutive monitor. Overall, both animal models suggested the strong anti-invasion and metastatic effects of SH.

In order to metastasize to new sites, cancer cells need to degrade down the ECM. MMPs and TIMPs act together to control ECM degradation [32, 33]. Therefore, MMPs and TIMPs changes are good markers of the metastatic activity of cancer cells. Research have shown that both MMPs and TIMPs are involved in breast cancer progression [24, 34]. Some studies even showed the different breast cancer subtypes contained different MMPs and TIMPs patterns [35, 36]. Here, we found that SH could inhibit MMP-9 and increased TIMP-1 and TIMP-2 both serum levels and cell expression levels in 4T1 mouse model. However, we only detected the same cell expression changes in MDA-MB-231-luc mouse model due to very low concentrations of these factors in serum. The decreased MMP-9 and increased TIMP-1 and TIMP-2 together indicated a change of metastatic activity after SH treatment in the two mouse models.

EMT is demonstrated to play a role in the development of invasion, metastasis and therapy resistance [15]. EMT is an essential process during embryo genesis and adult tissue repair and maintenance, with characteristics of loss of epithelial traits such as cell-cell adhesion and acquisition of mesenchymal properties such as acquisition of motility [15, 37]. It is accompanied by decrease epithelial markers such as E-cadherin, occludins and claudins, and increase mesenchymal markers such as N-cadherin and vimentin. E-cadherin is expressed at cell-cell adhesion junctions and required for the formation of epithelia during the embryo period and maintenance of epithelia homeostasis in adulthood [37]. Research have shown that low E-cadherin is related to higher tumor grade and stage, meanwhile, loss or genetic mutation of E-cadherin expression contributes cancer development [38]. However, E-cadherin reduction alone is not sufficient enough for EMT [38, 39]. The EMT also includes the acquisition of mesenchymal properties. We then started the investigation of EMT by examine the expression changes of the markers. Here, we reported that SH treatment increased E-cadherin while decreased N-cadherin in tumor specimens of 4T1 mouse model and lung specimens of MDA-MB-231-luc mouse model, suggesting SH could inhibit EMT, which might contribute its anti-invasion and anti-metastatic potentials.

Research have shown that cells that undergo EMT also acquire stem cell-like properties [16]. CSCs were discovered first in the hematopoietic system and later in human solid tumors such as breast cancer and brain cancer [40–42]. CSCs are a small population of neoplastic cells within a tumor with self-renewal capability and tumor initiating ability and are shown to play a role in the malignant process of tumors, such as drug resistance, invasion and metastasis [15]. EMT enables cancer cells the ability to metastasize as well as the ability to replicate in order to form new tumor foci [13, 16]. The pluripotent cell marker includes but not limits to CD44, OCT4, SOX2, Klf4 and Nanog. CD44, a cell-surface glycoprotein involved in cell adhesion and migration, is recognized as one of the important CSC markers, especially in mammary CSCs [43]. There are also some studies showing that Sox-2, but not OCT4 and Nanog, that plays an important role in breast CSCs [44]. Research has shown that the expression of Sox-2 in early stage of breast cancers [45]. Also, estrogen receptor α could regulate breast tumor-initiating cells targeting Sox-2 [46]. Our experiments demonstrated that SH down-regulated CD44 and Sox-2 expression in primary tumor specimens and metastatic lung specimens. The inhibition of CSC properties by SH was confirmed using *in vitro* mammosphere formation assay, which depends on the self-renewing ability of CSCs.

Microenvironment and cytokines related to tumor microenvironment are known to induce EMT [47]. Meanwhile, the factors play a role in the maintenance of post-EMT mesenchymal status once it is started [20]. SH is most famous for its treatment of rheumatic arthritis due to anti-inflammatory effects. In our research, when we removed organs from mouse, we found that SH decreased spleen volume and weight, especially in the 4T1 mouse model, which could be due to the partial immunosuppression of nude mice used in the MDA-MB-231 mouse model. Spleen is an organ involved in the inflammation response. So we hypothesized that the inhibition effect of SH on EMT could be contributed by its inhibition on the production of inflammatory factors. IL-6 is one of the important cytokines involved in the EMT process [19] and previous studies concerning SH also demonstrated that SH could inhibit IL-6 [48, 49]. Our results found that SH reduced IL-6 expression in tumor specimens and lung specimens. We then used both tumor cells and macrophage cells, two of the many different sources of IL-6, to detect the effect of SH on IL-6 production *in vitro*. We found that SH decreased IL-6 production in both tumor cells MDA-MB-231 and RAW 246.7. IL-6 concentration was too low to detect in 4T1 cells. This might suggest that the reduction of IL-6 expression in tumor specimens and lung specimens could be an integrated result as suppression of IL-6 secretion in both tumor cells and inflammatory cells *in vivo*. However, even if the inhibition effect of SH on both tumor cells and inflammatory cells were confirmed by *in vitro* experiment, the mechanism *in vivo* may not be as simple as it was *in vitro*. Il-6 is not only secreted to influence other cytokines, but also to function to influence its own production [25]. Besides, IL-6 production from tumor cells and other inflammatory cells influence each other. There were studies showing that IL-6 production from macrophage could induce IL-6 secretion from tumor cells [50]. What's more, in the detection of MMP and TIMP in serum samples of mouse models, we could find secretion changes in samples from MDA-MB-231 but at a very low level which required too much samples. In contrast, in the 4T1 mouse model, we detected relatively high concentrations of MMP and TIMP. The actual total serum cytokines came from both cancer cells and mouse inflammatory cells. For the MDA-MB-231 mouse model, because we detected the production of these cytokines of human MDA-MB-231 in the mouse model and thus the human cytokines of MMP and TIMP secretion might be diluted in mouse serum. This might explain why the serum secretion was so low and suggested that the cytokines were not only come from tumor cells or host cell *in vivo*. All this suggested the cytokines secretion in the mouse model was a complicated process. In all, comparing with the control group, the remarkable decrease effect of SH on spleen volume and spleen weight, as well as the reduction effect of IL-6 production, indicated that SH could inhibit breast cancer metastasis by suppressing EMT and CSC which was due to the anti-inflammation effect such as IL-6.

Although more studies focusing on the anti-cancer effect of SH need to be done, this research gives us a new sight into the clinically used drug SH beyond its normal anti-rheumatic arthritis effect, helping us to uncover more potential anti-cancer effects of SH.

## MATERIALS AND METHODS

### Drug and agents

SH was obtained from Zhengqing Pharmaceutical Group (Hunan, China). SH was dissolved in Dulbecco's modified Eagle's medium (DMEM) at 10 μmol/mL and filtered using 0.22 μm sterile filters (Millipore). Matrigel was purchased from BD Bioscience (Pasadena, CA, USA). Transwell chambers with 8 μm pore polycarbonate membrane filters (Corning, NY, USA) were used to explore the migration and invasion ability of 4T1 and MDA-MB-231 cells in response to SH. The ELISA kits of MMP-2 (CSB-E04676m for mouse; CSB-E04675h for human), MMP-9 (CSB-E08007m for mouse; CSB-E08006h for human), TIMP-1 (CSB-E08004m for mouse; CSB-E08003h for human), TIMP-2 (CSB-E07387m for mouse; CSB-E04733h for human) and IL-6 (CSB-E04639m for mouse; CSB-E04638h for human) were bought from Cusabio biotech (Wuhan, China).

The antibodies against E-cadherin, N-cadherin and Vimentin were obtained from Cell Signaling Technology (Beverly, MA, USA). The antibodies against MMP-9, TIMP-1, TIMP-2, Sox-2, β-actin and horse-radish peroxidase (HRP) conjugated anti-rabbit or mouse IgG were purchased from Santa Cruz Biotechnology (Santa Cruz, CA, USA). The antibodies for IL-6 and CD44 were bought from Abcom Biotechnology (Cambridge, MA, USA).

### Cell culture

Murine breast cancer cell line 4T1, murine macrophage cell line RAW246.7, and human breast cancer cell line MDA-MB-231 were obtained from Shanghai Institute of Cell Biology (Shanghai, PR China) in the Chinese Academy of Sciences. The bioluminescent cell line MDA-MB-231-luc was obtained in our previous experiments [51]. All cell lines were cultured in DMEM supplemented with 10% fetal bovine serum (FBS), 100 U/ml penicillin and 100 μg/ml streptomycin and incubated at 37°C in an incubator with 5% CO2 and saturated humidity.

### Wound healing assay

Wound healing assay was performed to assess the motility of cells exposed to SH. 4T1 and MDA-MB-231 cells were seeded in 6-well plates. When cells grew up to 90–100% confluence, the cell monolayer was scraped by a white pipette tip. Then the detached cells were washed with PBS and serum-free medium containing different concentrations of SH was added. Pictures were taken under an inverted microscopy (Leica, Germany) at 0, 12 and 24 h. The percentage of inhibition was normalized as the percentage of the untreated cells (100%).

### Transwell migration assay

Briefly, a total of 1 × 10^5^ 4T1 and MDA-MB-231 cells in 200 μL serum-free medium were added in the upper transwell chambers and treated with various concentrations of SH. The lower chambers were filled with 600 μL medium containing 10% FBS. After 24 h, the non-migratory cells on the upper surface of the chambers were removed by a cotton swab and the migrated cells were fixed in 4% paraformaldehyde and stained with 0.5% crystal violet. Then at least five randomly chosen fields were photographed under a microscope (Leica, Germany). Finally, the migrated cells were dissolved in 30% acetic acid and measured by a microplate reader (PerkinElmer, USA) at 570 nm. The percentage of inhibition was normalized as the percentage of the untreated cells (100%).

### Matrigel invasion assay

Matrigel was diluted 1:5 with DMEM. Transwell filters were coated with 50 μl the dilution. A total of 1 × 10^5^ 4T1 and MDA-MB-231 cells in 200 μL serum-free medium were added in the upper chamber and treated with various concentrations of SH. The lower chamber was filled with 600 μL medium containing 10% FBS. After incubation for 24 h, cells were treated the same as the procedures for the migration assay.

### Mammosphere formation assay

Mammosphere formation assay was performed as previously described [52]. Cells were treated with SH for 48 h and collected to seed into ultralow attachment plates (Corning, NY, USA) at a density of 4000 cells / well. Cells were cultured in serum-free DMEM/F12 medium supplemented with 20 ng/ml human epidermal growth factor (EGF), 10 ng/ml human basic fibroblast growth factor (bFGF), 2% B27 supplement and 1% N2 supplement. On day 10, the number of tumorspheres (diameter > 50 μm) in each well were evaluated using an inverted microscope.

### FACScan flow cytometer analysis

Cells were seeded in 6-well plates (Corning, NY) and treated with different concentrations of SH. Then cells were collected at the indicated time. Samples of at least 1 × 10^6^ cells were stained with anti-CD44-APC conjugate (103008, Biolegend) at 4 °C for 30 min. The cells were washed 3 times and then analyzed by flow cytometry.

### Western blot assay

Cells were treated with SH and lysed in RIPA. Proteins were collected and analyzed using a similar method as our previously reported study [5].

### Orthotopic breast tumor metastasis model

All animal procedures were performed according to the protocol approved by the Institutional Animal Care and Use Committee at Xi'an Jiaotong University. Four-week old female Balb/c strains of mice with body weights approximately 18–20 g were obtained from and housed in the Laboratory Animal Centre of Xi'an Jiaotong University. Each mouse was injected with 1 × 10^5^ 4T1 cells into the left second mammary fat pad (day 1). The mice were randomly distributed into three groups (*n* = 6) and intraperitoneally (i.p.) injected with 0.1 mL physiologic saline per 10 g body weight daily (control group) or SH at doses of 75 or 150 mg/kg of body weight, dissolved in physiologic saline (experimental groups) from the third day. Tumor sizes were measured twice or three times a week using calipers and tumor volumes were calculated according to the standard formula: width^2^ * length/2 and expressed as cm^3^. The mice were treated for four weeks. At the end of treatment, the mice were sacrificed and the organs and tumors of the mice were collected. Lung tissues were fixed in Bouin's solution for 24 h and taken pictures. Spleen volumes were calculated using the formula: length*width*height and expressed as cm^3^. The tissues were fixed and prepared for Hematoxylin and eosin (H&E) staining and immunohistochemistry (IHC) analysis.

### Experimental breast tumor metastasis model

Four-week old female BALB/c athymic nude mice with body weights approximately 18–20 g were purchased from Shanghai SLAC Laboratory Animal Co. Ltd. (Shanghai, China) and housed in the Laboratory Animal Centre of Xi'an Jiaotong University. After one week's acclimation, 1 × 10^6^ MDA-MB-231-luc cells in 100 μl PBS were injected into the tail vein of nude mice. On the day following injection, the mice were randomly assigned to three group (*n* = 6). Animals in the control group were i.p. injected with 0.1 mL physiologic saline per 10 g body weight daily and the treatment groups were injected i.p. with SH at doses of 75 or 150 mg/kg of body weight, dissolved in physiologic saline. Bioluminescence imaging (BLI) was performed weekly using luciferin and a Xenogen IVIS 2000 Luminal Imager to monitor lung metastasis for 4 weeks. Luciferin was stocked at a concentration of 15 mg/mL and was injected i.p. at a dose of 10 μL/g of mouse weight. Luminescence is expressed as photons/sec/ROI (region of interest). At the experimental endpoint, mice were euthanized and the organs were prepared for H&E staining and IHC analysis. For H&E analysis, the metastatic foci area was divided into five grades and numbered 1 to 5. The total numbers of each histologic section were added together as the index for lung or liver metastasis.

### IHC analysis

IHC analysis were performed as previous [5]. The intensity of the staining was scored as 1 (negative), 2 (weakly positive), 3 (moderately positive) or 4 (strongly positve). The extent of the staining was categorized as 1 (stained cells: 1–25%), 2 (26–50%), 3 (51–75%) or 4 (76–100%) for the tissues. The final staining score was the product of the intensity and the extent scores. Five images of random fields were taken from each specimen for quantitative analysis.

### Statistical analysis

All data are expressed as mean ± standard deviation (SD) from at least three independent experiments. The differences between the control and treatment groups were compared by *t*-test or ANOVA methods. All statistical analyses were performed using (SPSS Inc., Chicago, IL, USA). *P* values < 0.05 (*) or < 0.01 (^#^) were considered as statistically significant.

## SUPPLEMENTARY TABLES AND FIGURES



## Abbreviations

SH: sinomenine hydrochloride
EMT: epithelial to mesenchymal transition
CSC: cancer stem cell
ECM: extracellular matrix
MMP: matrix metalloproteinase
TIMP: tissue inhibitor of metalloproteases
ELISA: enzyme-linked immunosorbent assay
DMEM: Dulbecco's modified Eagle's medium
HRP: horse-radish peroxidase
FBS: fetal bovine serum
i.p.: intraperitoneally
H&E: hematoxylin and eosin
IHC: immunohistochemistry
SD: standard deviation.
