# Supplementary material for: Sinomenine hydrochloride inhibits breast cancer metastasis by attenuating inflammation-related epithelial-mesenchymal transition and cancer stemness
Source: Oncotarget. 2017 Jan 10;8(8):13560–74. doi: 10.18632/oncotarget.14593 (PMC5355120; doi:10.18632/oncotarget.14593)
Supplement: Supplementary file 1 [file oncotarget-08-13560-s001.pdf]

Sinomenine hydrochloride inhibits breast cancer metastasis by attenuating inflammation-related epithelial–mesenchymal transition and cancer stemness

Supplementary Materials

Supplementary Table 1: Hepatotoxicity and nephrotoxicity evaluation of SH on 4T1 mouse model

	Liver function		Renal function	
Treatment	AST(U/L)	ALT(U/L)	BUN(mmol/L)	CRE(μmol/L)
control	262.94 ± 58.89	39.03 ± 9.00	15.30 ± 2.65	10.89 ± 3.27
SH(75 mg/kg)	251.09 ± 45.78	32.06 ± 4.08	12.80 ± 1.99	9.78 ± 2.91
SH(150 mg/kg)	227.72 ± 51.72	32.30 ± 2.69	13.38 ± 1.31	8.89 ± 2.01

BALB/c mice bearing 4T1 mouse breast homograft were treated with physiologic saline or different doses of SH. Liver function was evaluated by AST and ALT. Renal function was evaluated by BUN and CRE. Data are represented as mean ± S.D. of three independent experiments. SH treated group compared with the untreated control group.

Supplementary Table 2: Hepatotoxicity and nephrotoxicity evaluation of SH on MDA-MB-231-luc mouse model

	Liver function		Renal function	
Treatment	AST(U/L)	ALT(U/L)	BUN(mmol/L)	CRE(μmol/L)
control	185.20 ± 14.70	50.53 ± 5.37	8.32 ± 1.24	12.67 ± 3.06
SH(75 mg/kg)	187.27 ± 36.10	49.27 ± 8.06	9.53 ± 1.43	11.00 ± 1.73
SH(150 mg/kg)	176.80 ± 16.80	46.73 ± 2.53	8.77 ± 1.55	11.17 ± 1.89

BALB/c mice was injected with MDA-MB-231-luc via tail vein and treated with physiologic saline or different doses of SH. Liver function was evaluated by AST and ALT. Renal function was evaluated by BUN and CRE. Data are represented as mean ± S.D. of three independent experiments. SH treated group compared with the untreated control group.

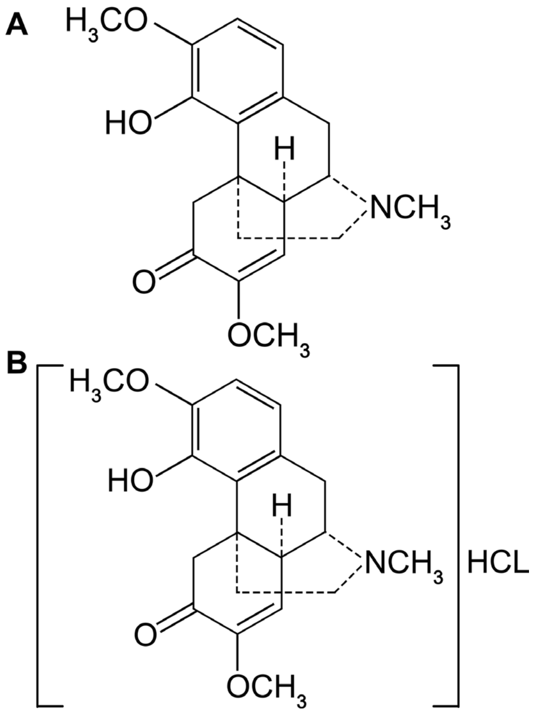

Supplementary Figure 1: Chemical structure of (A) sinomenine and (B) hydrochloride sinomenine (SH).

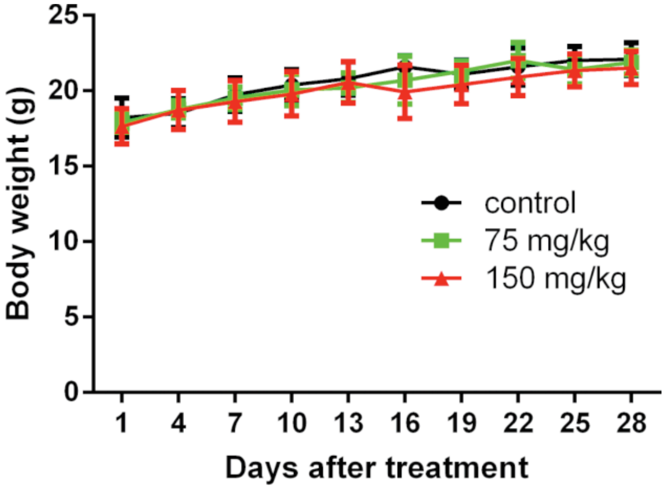

Supplementary Figure 2: BALB/c mice was injected with MDA-MB-231-luc via tail vein to establish the experimental mouse model. Body weight was measured during the treatment course.
